# Sociodemographic, clinical characteristics, and treatment patterns of endometrial cancer cases in Puerto Rico during the period 2009 to 2015: A retrospective study

**DOI:** 10.1371/journal.pone.0302253

**Published:** 2024-05-02

**Authors:** Yisel Pagán Santana, Maira Castañeda Ávila, Ruth Ríos Motta, Karen J. Ortiz Ortiz

**Affiliations:** 1 Department of Health Services Administration, School of Public Health, University of Puerto Rico Medical Sciences Campus, San Juan, Puerto Rico; 2 Division of Epidemiology, Department of Population and Quantitative Health Sciences, University of Massachusetts Chan Medical School, Worcester, Massachusetts, United States of America; 3 Division of Cancer Control and Population Sciences, University of Puerto Rico Comprehensive Cancer Center, San Juan, Puerto Rico; QUT Health: Queensland University of Technology Faculty of Health, AUSTRALIA

## Abstract

**Background:**

Over the past decades, the rising incidence rates of endometrial cancer have made it a significant public health concern for women worldwide. Treatment strategies for endometrial cancer vary based on several factors such as stage, histology, the patient’s overall health, and preferences. However, limited amount of research on treatment patterns and potential correlations with sociodemographic characteristics among Hispanics is available. This study analyzes the treatment patterns for patients diagnosed with endometrial cancer in Puerto Rico.

**Methods:**

A secondary database analysis was performed on endometrial cancer cases reported to the Puerto Rico Central Cancer Registry-Health Insurance Linkage Database from 2009 to 2015 (n = 2,488). The study population’s sociodemographic and clinical characteristics were described, along with an overview of the therapy options provided to patients receiving care on the island. Logistic regression models were used to evaluate the association of sociodemographic/clinical characteristics with treatment patterns stratified by risk of recurrence.

**Results:**

In our cohort, most patients were insured through Medicaid and had a median age of 60 years. Almost 90% of patients received surgery as the first course of treatment. Surgery alone was the most common treatment for low-risk patients (80.2%). High-risk patients were more likely to receive surgery with radiotherapy and chemotherapy (24.4%). Patients with Medicare insurance were five times (HR: 4.84; 95% CI: 2.45–9.58; p < 0.001) more likely to receive surgery when compared with patients insured with Medicaid. In contrast, those with private insurance were twice as likely to receive surgery (HR: 2.38; 95% CI: 1.40–4.04; *p* = 0.001) when compared to those with Medicaid.

**Conclusion:**

These findings provide insight into the treatment patterns for endometrial cancer in Puerto Rico and highlight the importance of considering factors such as disease risk when making treatment decisions. Addressing these gaps in treatment patterns can contribute to effective management of endometrial cancer.

## Introduction

One of the most common cancers in women is endometrial cancer, which affects the uterine lining [1]. The incidence and prevalence of endometrial cancer can vary between different populations. In Puerto Rico, it is the fourth most diagnosed cancer in women [2]. According to the most recent cancer report, there has been an ongoing annual rise in endometrial cancer incidence rates of 4.7% in the Puerto Rican population between 2000 and 2018. Although the incidence of endometrial cancer in Puerto Rico is lower than in the United States [3], highlighting the rise in incidence observed in recent years is crucial to underscore the significance of this study. Additionally, differences in access to healthcare services can impede early diagnosis and proper treatment, leading to poorer outcomes [4].

After a cancer diagnosis, correct treatment can potentially improve the chances of remission and survival. For endometrial cancer, the treatment choice may depend as much on the grade, stage, and histology as the desire to maintain fertility [5]. Standard treatment consists of total hysterectomy with bilateral salpingo-oophorectomy (BSO) with or without lymph node dissection or the addition of adjuvant therapy. The decision to provide adjuvant treatment is based on the risk of the disease. Low-risk metastatic tumors can be treated with surgery alone, while in patients with high-risk metastatic tumors, additional adjuvant therapy is preferred [6].

Evidence shows differences in treatment patterns depending on specific clinical and socioeconomic factors [7]. Although some differences in treatment based on stage, grade, and tumor type can be justified, other differences in treatment, like being older, are not supported by clinical consensus [8]. Existing differences in cancer treatment among medically indigent individuals and those enrolled in Medicaid are also found in the literature [9, 10]. Therefore, assessing the effect of these observed disparities in endometrial cancer care is imperative.

Puerto Rico is characterized by a primarily Hispanic population (98.9%), a high poverty rate (40.5%), and increased health insurance coverage (94.3%) [11]. Although Puerto Rico has a high rate of insured individuals, studies have pointed out health disparities in quality of care and clinical differences that could influence endometrial cancer outcomes [12]. Little is known about the treatment patterns of patients diagnosed with endometrial cancer in Puerto Rico. To our knowledge, no previous study has comprehensively summarized the therapy options provided to the rising number of endometrial cancer patients in Puerto Rico. This study describes the population profile according to the sociodemographic and its clinical characteristics and the patterns of endometrial cancer treatment.

## Materials and methods

### Data source

Using the Puerto Rico Central Cancer Registry-Health Insurance Linkage Database (PRCCR-HILD), this secondary data analysis employed a retrospective cohort design. Although the study does not involve prospective follow-up, it captures information over a period (2009–2015), allowing for an examination of the association of patient characteristics and first course treatment received. The Puerto Rico Central Cancer Registry (PRCCR) is part of the Centers for Disease Control and Prevention (CDC) National Program of Cancer Registries (NPCR). It adheres to data processing standards for the Surveillance, Epidemiology, and End Results (SEER) Program and North American Association of Central Cancer Registries (NAACCR) coding standards. All healthcare facilities in Puerto Rico are required by law to report cancer cases to the PRCCR, which obtains clinical and demographic data from hospitals, outpatient clinics, pathology laboratories, and radiotherapy/chemotherapy sites across the island. Over the years, the PRCCR has enhanced its data collection for cancer cases using electronic reporting, consistently achieving a completeness rate of over 95% for all cases annually since 2010. This accomplishment has enabled the PRCCR to collaborate and function as a valuable source of information in significant local and international scientific publications [2]. The PRCCR maintains up-to-date information through linkages with other databases, such as death certificates and health insurance claims databases. Since 2008, cancer cases have been linked to the health insurance claims files (HILD), containing information for nearly 90.0% of Puerto Rico cancer cases [13]. Endometrial cancer cases were obtained from the PRCCR-HILD, which has met the completeness and data quality standards [14]. Since our database consisted of de-identified data, this study was considered exempt from review by the University of Puerto Rico Institutional Review Board.

### Study population

**Fig 1:**
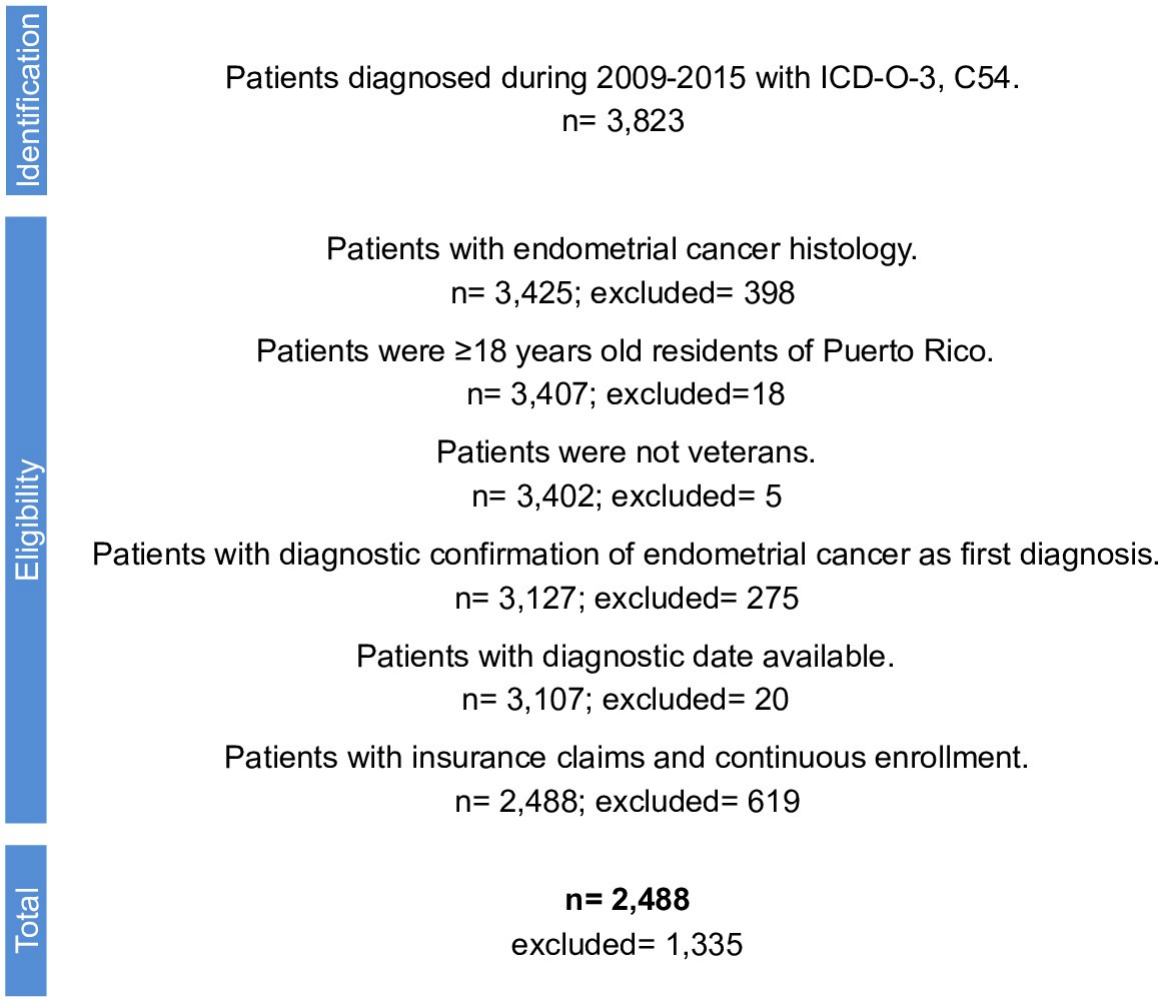
Flow diagram of the case selection process.

The study population comprised women diagnosed with endometrial cancer from January 1, 2009, to December 31, 2015 (Fig 1).

### Inclusion criteria

This study included residents of Puerto Rico with a first diagnosis of endometrial cancer as defined by the International Classification of Diseases for Oncology, Third Edition (ICD-O-3), code C54x. Type I histology included codes 8010, 8140, 8380, 8381, 8382, 8383, 8480, 8481, 8482, 8560 and 8570. Type II histology included codes 8020, 8021, 8041, 8050, 8070, 8071, 8072, 8260, 8310, 8323, 8441, 8460, 8461, 8574, 8980, 8951, 8950 [15–17]. Only cases of endometrial cancer 18 years or older with diagnostic confirmation were included in the study.

### Exclusion criteria

Patients with unknown diagnostic confirmation, incomplete date of diagnosis (month/year), unknown age, and without continuous health insurance enrollment were excluded. We refer to a patient with continuous enrollment as a patient who was actively enrolled in or covered by any health insurance included in our analysis (Medicaid, Medicare, Medicare-Medicaid, or private). We decided that people without continuous enrollment for at least a year in their health plan would be excluded from the analysis. We applied this exclusion criterion to ensure the reliability and integrity of the data, as individuals with enrollment gaps may have incomplete or inconsistent records, making it difficult to accurately assess the variables of interest. Excluding such individuals helped us maintain the internal validity of the study by focusing on a more homogeneous and well-defined study population. In addition, cases reported to the PRCCR-HILD identified only by the death certificate or autopsy were excluded from the study. Patients from the Veterans Health Administration were also excluded.

### Operational definitions of variables

#### The first course of treatment

The first course of treatment was classified as no treatment, surgery only, surgery with radiotherapy, surgery with chemotherapy, surgery with chemotherapy and radiotherapy, radiotherapy and chemotherapy, chemotherapy only, and radiotherapy only. Also, treatment was grouped according to modality: surgery only, surgery with adjuvant therapy, or chemotherapy/ radiotherapy only. The adjuvant treatment therapies considered were radiotherapy and chemotherapy.

#### Sociodemographic characteristics

Other patient characteristics evaluated in the study were age at diagnosis and socioeconomic position estimated using the CDC/Agency for Toxic Substances and Disease Registry (ATSDR) Social Vulnerability Index [18]. Marital status at the diagnosis was classified as married (including common law or domestic partner), unmarried (never married, separated, divorced, or widowed), and unknown. Health insurance types included: Medicaid, Medicare, dually eligible for both Medicare and Medicaid, and private. Health regions were divided as stipulated by the Puerto Rico Department of Health (DSPR, by its acronym in Spanish) (S1 Table) [19].

#### Clinical characteristics

Additional clinical variables were also incorporated into the study. To assess the prevalence of comorbidities in our cohort, we used an adaptation of the Charlson comorbidity index (CCI) developed by Klabunde and colleagues [20]. The Charlson comorbidity index was classified as 0, 1, and ≥ 2. The ICD-O-3 codes classified histology types of endometrial carcinoma cases into Type I and II. The tumor stage was classified according to the International Federation of Gynecology and Obstetrics (FIGO, by its acronym in French) staging and summarized as stage I, II, III, IV, and unknown. Tumor grade was classified as grade 1 (well differentiated—low grade), grade 2 (moderately differentiated—intermediate grade), grade 3 (poorly differentiated/ undifferentiated—high grade), or unknown grade (grade cannot be assessed—undetermined grade). Grade 3 includes those tumors that are poorly differentiated (or grade 3) and those that are undifferentiated (known as grade 4) following FIGO grading system.

#### Risk of recurrence

Finally, an adaptation of the risk criteria from the *Adjuvant Chemoradiotherapy Versus Radiotherapy Alone in Women with High-Risk Endometrial Cancer* (PORTEC-3) trial was used to assign patients into three groups: low-risk, medium-risk, and high risk (S2 Table). To assess myometrial invasion, we used the FIGO staging system where stages I, I NOS, and IA are grouped as <50.0% invasion and all other stages as ≥50.0% [21]. The criteria also incorporated lymphovascular invasion, but it was not feasible to include it due to the unavailability of the information for all patients.

### Statistical methods

Descriptive statistics and frequency analyses were used to describe the variables of interest. To evaluate the receipt of treatment, differences in treatment patterns, and the independent variables of interest, the Chi-square (χ2) test was used. To analyze the relationship between treatment patterns and sociodemographic characteristics, logistic regression models were employed. These models provided estimates for unadjusted odds ratios (ORs), adjusted odds ratios (AORs), and their corresponding 95% confidence intervals (CIs).

Two analyses were conducted to examine treatment modalities. The first analysis encompassed surgical interventions either alone or in combination with adjuvant treatment. The second analysis focused on the binary categorization of whether surgery was received or not. For the latter, stage 4 or unknown stage patients were excluded due to the low frequency of surgical interventions in these cases (excluded n = 464). We looked at several models that considered different covariates and stratified data. The Bayesian Information Criterion (BIC) was used to assess and select the most suitable model for the data.

The chosen model for the first analysis, identified as the most suitable due to its lowest BIC, was adjusted for marital status, type of health insurance, comorbidities, and health region. Additionally, it was stratified according to disease risk. This model’s definition of curative treatment included surgery alone or surgery with adjuvant therapy. Since chemotherapy or radiation therapy alone are typically considered palliative treatments, they were not included in the analysis.

The optimal model for the second analysis was adjusted for age, marital status, insurance, comorbidities, region, grade, stage and histology. Unlike the first analysis this analysis was not stratified. Statistical analysis was conducted using the Stata statistical software (Release 17. College Station, TX: StataCorp LLC).

## Results

### Demographic & clinical characteristics

A total of 2,488 patients with endometrial cancer diagnosed in Puerto Rico between January 1, 2009, and December 31, 2015, were included in our study (Fig 1). The median age was 60 years. Most patients were insured through Medicaid (33.5%) and had CCI scores between 0 and 1 (68.4% and 20.3%, respectively) (Table 1). Nearly all endometrial cancer histology was type I (88.7%), while 11.3% were type II. Around 1,618 (65.0%) were diagnosed in stage I, while 870 (35.0%) were diagnosed at later stages. Close to 80.0% of the tumors were grade 1 or 2. Also, in our population, more than half of the patients were classified at medium risk of recurrence (56.2%).

**Table 1:** Clinical and sociodemographic characteristics of the patient sample.

Variable	Total	
	n	%
**Age**		
<50	479	19.3
50–59	677	27.2
60–69	824	33.1
≥70	508	20.4
**CDC/ATSDR Social Vulnerability Index** *		
1 QT	839	33.7
2 QT	830	33.4
3 QT	819	32.9
**Marital status**		
Married	1,236	49.7
Unmarried	1,184	47.6
Unknown	68	2.7
**Insurance type**		
Medicaid	834	33.5
Medicare	553	22.2
Medicare/Medicaid	328	13.2
Private	773	31.1
**Health regions**		
North	302	12.1
Central	379	15.2
Southeast	406	16.3
East	72	2.9
West	381	15.3
Northeast	492	19.8
South	456	18.3
**Comorbidity Index**		
0	1,701	68.4
1	504	20.3
≥2	283	11.4
**Year of diagnosis**		
2009	214	8.6
2010	293	11.8
2011	323	13.0
2012	376	15.1
2013	408	16.4
2014	420	16.9
2015	454	18.3
**Stage at diagnosis**		
I	1,618	65.0
II	174	7.0
III	232	9.3
IV	105	4.2
Unknown	359	14.4
**Myometrial invasion**		
<50%	1,235	49.6
≥50%	1,253	50.4
**Histology type**		
Type I	2,207	88.7
Type II	281	11.3
**Grade**		
1	1,181	47.5
2	777	31.2
3	335	13.5
Unknown	195	7.8
**Disease risk of recurrence**		
Low risk	572	23.0
Medium risk	1,399	56.2
High risk	517	20.8

* According to variable RPL_THEME1.

Abbreviations: n, represents the total number of cases in the population; %, percentage; CDC/ATSDR, Centers for Disease Control and Prevention and Agency for Toxic Substances and Disease Registry; QT, quartile; Medicaid/Medicare, dually eligible.

### Treatment patterns

About 2,194 (88.2%) of patients received surgery as a first course of treatment. In more than half of the patients who underwent surgery, the most frequently performed procedure was total hysterectomy with BSO (61.8%) (Table 2). The most common treatment modality was surgery only (52.9%), followed by surgery plus radiotherapy (18.5%).

**Table 2:** Treatment patterns and types of surgery performed on the study population.

Variable	Category	Total	
		n	%
**First course of treatment**	None	164	6.6
	Surgery Only	1,316	52.9
	Surgery + Radiotherapy	461	18.5
	Surgery + Radiotherapy/Chemotherapy	275	11.1
	Surgery + Chemotherapy	142	5.7
	Radiotherapy	56	2.3
	Radiotherapy + Chemotherapy	51	2.1
	Chemotherapy	23	0.9
**Type of surgery**	No surgery	294	11.8
	TH with BSO	1,537	61.8
	Modified radical or extended hysterectomy	222	8.9
	TH without BSO	114	4.6
	Hysterectomy, NOS	250	10.1
	Local tumor resection	65	2.6
	Pelvic exenteration	6	0.2
**Treatment modality** *	Only surgery	1,316	56.6
	Surgery plus adjuvant therapy	878	37.8
	Chemotherapy and/or radiotherapy	130	5.6

*Patients without treatment were excluded. Values are presented as n (column %).

Abbreviations: TH- total hysterectomy, BSO- bilateral salpingo-oophorectomy, NOS- not otherwise specified.

**Fig 2:**
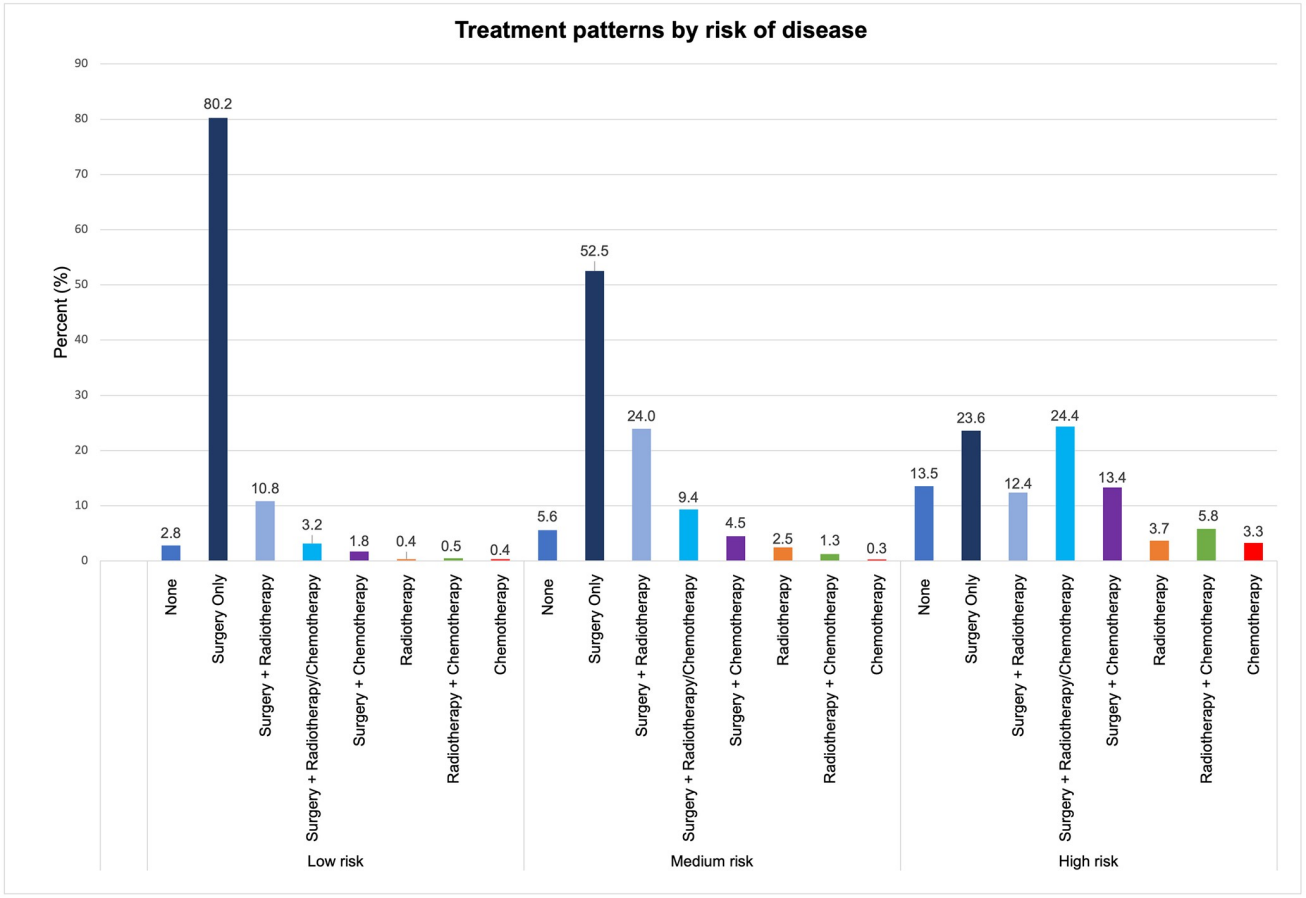
Treatment patterns by the risk of disease.

Almost all patients with a low risk of endometrial cancer received surgery alone (80.2%) (Fig 2). Surgery with radiotherapy and chemotherapy was the most frequent treatment for high-risk patients (24.4%). Those who received chemotherapy alone or combined with surgery or radiation were mainly at high risk.

After adjusting for marital status, medical insurance, comorbidities, and health region, women at medium-risk disease with Medicare (AOR: 0.61, [95% CI: 0.45–0.84], *p* = 0.002) or private insurance (AOR: 0.65, [95% CI: 0.48–0.89], *p* = 0.006) were less likely to have surgery with adjuvant therapy compared to Medicaid patients (Table 3) (n = 2, 194). In addition, women with high-risk disease insured with Medicaid-Medicare were twice as likely to receive surgery with adjuvant therapy compared to Medicaid patients (AOR: 2.23, [95% CI: 1.02–4.87], *p* = 0.045). Differences in treatment according to the region were also found. Women at medium risk on the southeast, west, or south of the island were more likely to receive surgery with adjuvant therapy when compared with women in the north region (63.0%, [95% CI: 1.05–2.52], *p* = 0.030, 64.0%, [95% CI: 1.06–2.53], *p* = 0.025, 72.0%, [95% CI: 1.13–2.62], *p* = 0.012, respectively). On the other hand, women from the west region at high risk were three times (up to 8 times) more likely to receive surgery with adjuvant therapy when compared to the north region (AOR: 3.31, [95% CI: 1.31–8.36], *p* = 0.011).

**Table 3:** Crude and adjusted models by curative treatment: Surgery only [Ref] vs. surgery with adjuvant therapy.

Variable	Low Risk				Medium Risk				High Risk			
	Crude		Adjusted		Crude		Adjusted		Crude		Adjusted	
	OR (95% CI)	*p*	OR (95% CI)	*p*	OR (95% CI)	*p*	OR (95% CI)	*p*	OR (95% CI)	*p*	OR (95% CI)	*p*
**Marital status**												
Unmarried	1.00 [Ref]											
Married	0.90 (0.57–1.41)	0.640	0.90 (0.57–1.45)	0.674	0.90 (0.72–1.13)	0.361	0.90 (0.72–1.14)	0.389	1.00 (0.65–1.55)	0.986	0.99 (0.63–1.55)	0.967
Unknown	-	-	-	-	0.40 (0.18–0.89)*	0.025	0.39 (0.17–0.89)*	0.024	0.15 (0.02–1.51)	0.108	0.18 (0.02–1.83)	0.147
**Insurance type**												
Medicaid	1.00 [Ref]											
Medicare	0.34 (0.10–1.16)	0.085	0.38 (0.11–1.37)	0.141	0.63 (0.46–0.85)*	0.002	0.61 (0.45–0.84)*	0.002	1.21 (0.69–2.10)	0.503	1.21 (0.68–2.15)	0.510
Medicare-Medicaid	1.27 (0.48–3.33)	0.634	1.40 (0.52–3.83)	0.506	0.87 (0.61–1.24)	0.434	0.82 (0.57–1.18)	0.280	2.44 (1.15–5.16)*	0.020	2.23 (1.02–4.87)*	0.045
Private	0.71 (0.44–1.15)	0.162	0.76 (0.46–1.26)	0.289	0.63 (0.47–0.84)*	0.002	0.65 (0.48–0.89)*	0.006	1.21 (0.67–2.18)	0.521	1.07 (0.58–1.99)	0.820
**Health Region**												
North	1.00 [Ref]											
Central	0.82 (0.33–2.04)	0.673	0.85 (0.34–2.14)	0.737	1.29 (0.84–1.99)	0.243	1.37 (0.88–2.11)	0.161	1.34 (0.61–2.96)	0.466	1.43 (0.64–3.19)	0.385
Southeast	0.76 (0.32–1.80)	0.535	0.77 (0.32–1.84)	0.558	1.66 (1.07–2.56)*	0.023	1.63 (1.05–2.52)*	0.030	2.08 (0.90–4.78)	0.085	2.08 (0.90–4.83)	0.088
East	1.08 (0.25–4.54)	0.922	1.35 (0.31–5.89)	0.688	1.68 (0.83–3.42)	0.151	1.77 (0.87–3.62)	0.118	3.56 (0.69–18.48)	0.130	3.92 (0.74–20.71)	0.107
West	0.67 (0.26–1.70)	0.395	0.69 (0.27–1.78)	0.443	1.59 (1.04–2.45)*	0.033	1.64 (1.06–2.53)*	0.025	3.48 (1.40–8.68)*	0.007	3.31 (1.31–8.36)*	0.011
Northeast	0.62 (0.24–1.58)	0.320	0.64 (0.25–1.67)	0.363	1.21 (0.79–1.84)	0.379	1.30 (0.85–1.98)	0.230	1.66 (0.79–3.47)	0.179	1.77 (0.83–3.75)	0.139
South	1.16 (0.51–2.62)	0.728	1.10 (0.48–2.52)	0.819	1.72 (1.14–2.62)*	0.011	1.72 (1.13–2.62)*	0.012	1.36 (0.61–3.04)	0.458	1.48 (0.64–3.38)	0.358
**Comorbidity Index**												
0	1.00 [Ref]											
41	1.07 (0.60–1.92)	0.816	1.16 (0.64–2.11)	0.631	0.89 (0.68–1.18)	0.418	0.90 (0.68–1.19)	0.460	0.86 (0.50–1.47)	0.572	0.85 (0.48–1.48)	0.561
> = 2	0.58 (0.17–1.98)	0.385	0.68 (0.18–2.52)	0.567	1.15 (0.82–1.62)	0.422	1.21 (0.84–1.74)	0.312	1.11 (0.57–2.14)	0.756	0.93 (0.46–1.86)	0.833

*Statistically significant. Logistic regression was stratified by disease risk while adjusting by marital status, type of medical insurance, comorbidities, and health region. Low risk: <60 years with grade < = 2, myometrial invasion <50%, and type I histology. Medium risk: >60 years or; grade < = 2, myometrial invasion >50% and histology type I or; grade > = 3, myometrial invasion <50% and type I histology. High risk: All type II histology or; grade > = 3, myometrial invasion >50%, and type I histology (n = 2,194).

Abbreviations: Ref, reference variable; OR, odds ratios; CI, confidence intervals.

Patients with stage IV or unknown stages were respectively 83.0% and 90.0% less likely to receive surgery when compared to stage I. Therefore, for this additional analysis, women in stage IV and unknown stages were excluded since receiving surgical treatment is not indicated [5] (n = 2, 024). Our findings demonstrate that women aged 70 and older are 48.0% less likely to undergo surgery, although this observation was marginally significant (AOR: 0.52, [95% CI: 0.26–1.03], *p* = 0.062). Moreover, patients with Medicare insurance were five times more likely to receive surgery when compared with Medicaid. In contrast, those with private insurance were twice as likely when compared with Medicaid (HR: 4.84; 95% CI: 2.45–9.58; p < 0.001; Private AOR: 2.38, [95% CI: 1.40–4.04], *p* = 0.001) (S3 Table).

The same analysis demonstrated that patients with a comorbidity score ≥2 were 48.0% less likely to receive surgery compared to patients with a 0-comorbidity score (AOR: 0.52, [95% CI: 0.30–0.92], *p* = 0.024). Patients from the central region were five times more likely to undergo surgery than those from the north region (AOR: 5.28, [95% CI: 2.04–13.67], *p* = 0.001). Likewise, those from the south and southeast regions had more possibilities of receiving surgical treatment (South AOR: 1.99, [95% CI: 1.02–3.88], *p* = 0.044; Southeast AOR: 2.61, [95% CI: 1.23–5.52], *p* = 0.012). Those with unknown tumor grade had a 79.0% lower probability of surgery than grade 1 tumors (AOR: 0.21, [95% CI: 0.11–0.42], *p*< 0.001) (S3 Table).

## Discussion

Our study revealed that most patients underwent surgery as their initial treatment, with total hysterectomy BSO being the most performed procedure. Total hysterectomy with BSO, which involves removing the uterus, cervix, fallopian tubes, and ovaries, is the standard treatment for endometrial cancer [22]. In patients diagnosed with stage I disease, surgery, typically including a hysterectomy with BSO, is the primary treatment approach in the United States [23]. Adjuvant therapy may be combined with surgery for some patients depending on their age (recommended for those > 60 years) and the risk of recurrence or metastasis [5, 24]. However, it is not recommended for low-risk patients as it may decrease their quality of life and increase morbidity, as some studies suggest [25, 26].

### Differences in treatment by clinical factors

As anticipated, our findings indicate that the treatment strategies become more aggressive as the severity of the disease increases. Patients with low-risk disease may be treated with surgery alone, while those with medium to high-risk disease may benefit from a combination of surgery, radiotherapy, and chemotherapy. According to cancer treatment statistics in the United States, the predominant treatment method for patients with early-stage (I-II) endometrial cancer involves surgery alone or combined with radiation therapy. Meanwhile, surgery and chemotherapy with or without radiation are preferred for most stage III patients (70%) [23]. These results underscore the significance of customizing the treatment plan based on patient characteristics such as the cancer type, tumor grade, and disease stage.

Although there is no definitive medical consensus regarding the best treatment for patients with high-risk disease, external beam radiation therapy has been widely accepted as the standard adjuvant treatment [27]. Surgery with radiotherapy and chemotherapy was the most common treatment pattern observed in our high-risk disease population. The randomized PORTEC-3 trial concluded that combining adjuvant therapies for high-risk endometrial cancer does not significantly improve overall survival. However, when administered together, they could enhance 5-year failure-free survival compared to radiation therapy alone. Therefore, the benefits and risks of adjuvant therapy should be evaluated individually [27].

Our cohort showed that patients receiving chemotherapy alone were at high-risk-disease. In advanced stage/recurrent endometrial cancer, chemotherapy, including carboplatin/paclitaxel, is the primary adjuvant treatment [5]. Chemotherapy can also be used as a neoadjuvant treatment to shrink the tumor before surgery. Although not evaluated in our study, this strategy has increased in use as it has been shown to reduce perioperative morbidity while offering similar overall survival [28]. Moreover, recent studies advocate for the assessment of molecular characterization of endometrial cancer to investigate innovative options to manage high-risk disease, such as immunotherapy [29].

### Differences in treatment by sociodemografic factors

Our findings also shed light on the differences in treatment patterns depending on patients’ insurance type, region, and age. As our results show, insurance status appears to play a role in the receipt of surgical treatment, with patients with Medicare insurance having the highest likelihood of receiving surgery compared to those with other types of insurance. In a Uterine Cancer Evidence Review Conference report, insurance-mediated disparities were also described [7]. For example, in one of the studies mentioned, the authors found that patients without insurance or with Medicaid were less likely to have minimally invasive surgery than women with private insurance [30]. Similarly, a study that analyzed ten types of cancer found that Medicaid participants and those uninsured tended to have advanced stages, were less likely to receive cancer surgery and radiation therapy, and had poorer survival rates [31].

The likelihood of receiving surgical treatment under different types of insurance, particularly those that are government-funded, is an important issue. Medicare, a federal health insurance program in the United States primarily for people aged 65 and older, has certain features that can influence the likelihood that people under this coverage will receive surgical treatment. First, it is important to mention that Medicare provides comprehensive coverage for a variety of medical services, including surgical procedures [32]. This broader coverage could potentially improve access to surgical treatments for those enrolled in Medicare compared to people ensured by Medicaid who may have more limited coverage [33]. Moreover, there appears to be an association between geographical location and the likelihood of receiving surgery, as patients from the island’s central region were more likely to undergo surgical treatment than those from the northern region. Notably, the north health region comprises the municipalities of Lares, Utuado, Ciales, and Morovis, characterized by remote areas and low socioeconomic status [34]. Consequently, this disparity may stem from differences in socioeconomic factors, healthcare infrastructure, and the accessibility of surgical services. Other factors that could explain healthcare access issues despite insurance coverage include limited transportation methods, geographic distances, sociocultural barriers, and availability of services. Therefore, as stated by the Institute of Medicine (2003), to reduce these disparities, it is necessary to promote coordinated medical care so that patients with lower income, from distant areas or enrolled in publicly funded plans have the same access to medical care as a patient with other types of insurance [35].

A population-based study using the SEER database found that women ≥ 65 years with endometrial cancer receive less surgical treatment and, as a result, have poorer survival [36]. However, it has been demonstrated that minimally invasive surgery is still the most adequate approach for treating endometrial cancer in patients ≥ 65 years [37]. Additional research confirms that hysterectomy via minimally invasive surgery offers a significant survival benefit for older adults [38]. Therefore, age should not be an obstacle to receiving adequate treatment. However, our results revealed that our older age population has a lower probability of receiving surgery. This finding raises important considerations, especially given the predominantly elderly composition of our population, indicating the need for additional research and possible intervention. Continued research and collaboration with healthcare professionals, policymakers, and the older population themselves will be crucial in designing effective strategies to enhance surgical care accessibility and ensure that age does not become a limiting factor in receiving necessary medical interventions.

### Strengths and limitations

There are some limitations to our study that are related to the data we used. Specifically, our database does not provide information on patient income; therefore, we used the CDC/ATSDR Social Vulnerability Index as a substitute for assessing patients’ socioeconomic status. Since not all patients had information about the lymphovascular invasion, this variable could not be incorporated into the risk assessment. Additionally, since our database uses insurance claims, those uninsured or from the Veterans Health Administration were excluded from the analysis. Therefore, for these populations, conclusions cannot be made. Also, there are factors beyond the relationship between health insurance type and treatment patterns observed that should be considered. For example, even if age is included in the risk variable, it does not account for the age threshold required to qualify for Medicare insurance (i.e., being over 65 years old). Since Medicare is associated with being older, it could potentially influence the treatment patterns in Medicare patients, representing another potential limitation of the study. However, as far as we know, this is the first assessment of treatment patterns for endometrial cancer in Puerto Rico. Utilizing PRCCR-HILD, we investigated non-clinical factors that may influence receiving appropriate cancer treatment. Therefore, this study provides essential information for enhancing health outcomes by recognizing socioeconomic barriers that could affect standard cancer care.

## Conclusion

In conclusion, this is the first study using a large population-based cohort of endometrial cancer patients in Puerto Rico to describe the treatment provided to our patients. Overall, these findings highlight the complex interplay between clinical, social, and demographic factors in determining treatment decisions for endometrial cancer patients. The associations observed in results based on health insurance, age, or residence underscore unacceptable disparities. Treatment decisions ought to be primarily influenced by clinical factors such as stage, grade, and patients’ performance. This study reveals that medical treatment decisions are linked not only to clinical factors but also to social and demographic aspects, bringing attention to potential inequalities. Consequently, this marks the initial phase in unraveling the intricate explanations associated with these findings. They also underscore the importance of ensuring access to high-quality, evidence-based care for all patients, regardless of their insurance status or other demographic characteristics. Future publications may discuss the relationship between treatment patterns and their potential effects on survival, contributing valuable insights to the field of public health and our population.

## Supporting information

S1 Table Health regions as stipulated by Puerto Rico Department of Health. (PDF)

S2 Table Risk of recurrence classification based on PORTEC-3 trial criteria. (PDF)

S3 Table Adjusted models by surgery. (PDF)
