# Supplementary material for: Sociodemographic, clinical characteristics, and treatment patterns of endometrial cancer cases in Puerto Rico during the period 2009 to 2015: A retrospective study
Source: PLoS One. 2024 May 2;19(5):e0302253. doi: 10.1371/journal.pone.0302253 (PMC11065223; doi:10.1371/journal.pone.0302253)
Supplement: S1 Table — (PDF) [file pone.0302253.s001.pdf]

S1. Health regions as stipulated by Puerto Rico Department of Health

Health region	Municipality
North	Arecibo, Hatillo, Camuy, Quebradillas, Barceloneta, Florida, Manatí, Vega Baja, Morovis, Ciales, Utuado and Lares.
Central	Bayamón, Cataño, Toa Baja, Toa Alta, Dorado, Vega Alta, Naranjito, Corozal, Orocovis, Barranquitas and Comerío.
Northeast	San Juan, Guaynabo, Trujillo Alto, Carolina, Canóvanas and Loíza.
East	Río Grande, Luquillo, Fajardo, Ceiba, Vieques and Culebra.
Southeast	Caguas, Aguas Buenas, Gurabo, Juncos, Las Piedras, Naguabo, Humacao, Yabucoa, Maunabo, San Lorenzo, Cayey, Aibonito and Cidra.
South	Ponce, Adjuntas, Jayuya, Guánica, Yauco, Guayanilla, Peñuelas, Juana Díaz, Villalba, Coamo, Santa Isabel, Salinas, Guayama, Arroyo and Patillas.
West	Aguadilla, Isabela, San Sebastián, Moca, Aguada, Rincón, Añasco, Las Marías, Maricao, Mayagüez, Hormigueros, San Germán, Sabana Grande, Cabo Rojo and Lajas.
