# Supplementary material for: Sociodemographic, clinical characteristics, and treatment patterns of endometrial cancer cases in Puerto Rico during the period 2009 to 2015: A retrospective study
Source: PLoS One. 2024 May 2;19(5):e0302253. doi: 10.1371/journal.pone.0302253 (PMC11065223; doi:10.1371/journal.pone.0302253)
Supplement: S2 Table — (PDF) [file pone.0302253.s002.pdf]

S2. Risk of recurrence classification based on PORTEC-3 trial criteria.

Classification	Criteria
Low risk	<60 years with grade <=2, myometrial invasion <50% and type I histology
Medium risk	>60 years or;<br>grade <= 2, myometrial invasion >50% and histology type I or;<br>grade >= 3, myometrial invasion <50% and type I histology
High risk	All type II histology or;<br>grade >= 3, myometrial invasion >50% and type I histology
