# Supplementary material for: Sociodemographic, clinical characteristics, and treatment patterns of endometrial cancer cases in Puerto Rico during the period 2009 to 2015: A retrospective study
Source: PLoS One. 2024 May 2;19(5):e0302253. doi: 10.1371/journal.pone.0302253 (PMC11065223; doi:10.1371/journal.pone.0302253)
Supplement: S3 Table — (PDF) [file pone.0302253.s003.pdf]

S3. Adjusted models by surgery.

Variable	Surgery (No [Ref.]/Yes)		
	Adjusted		
	Odds ratio	95% CI	*p*
**Age**			
<50	1.00 [Ref]		
50-59	1.29	0.68-2.45	0.441
60-69	1.02	0.55-1.87	0.953
70+	0.52	0.26-1.03	0.062
**Marital status**			
Unmarried	1.00 [Ref]		
Married	1.18	0.79- 1.75	0.43
Unknown	-	-	-
**Insurance type**			
Medicaid	1.00 [Ref]		
Medicare	4.84	2.45-9.58	<0.001
Medicare-Medicaid	1.36	0.75-2.43	0.309
Private	2.38	1.40-4.04	0.001
**Health Region**			
North	1.00 [Ref]		
Central	5.28	2.04-13.67	0.001
Southeast	2.61	1.23-5.52	0.012
East	1.40	0.48-4.08	0.538
West	1.20	0.63-2.27	0.574
Northeast	1.30	0.70-2.43	0.401
South	1.99	1.02-3.88	0.044
**Comorbidity Index**			
0	1.00 [Ref]		
1	1.06	0.62-1.78	0.841
>=2	0.52	0.30-0.92	0.024
**Stage***			
I	1.00 [Ref]		
II	0.98	0.50-1.94	0.945

III	0.77	0.44-1.34	0.354
**Histology**			
Type I	1.00 [Ref]		
Type II	0.86	0.47-1.57	0.630
**Grade**			
1	1.00 [Ref]		
2	1.02	0.62-1.66	0.950
3	0.57	0.32-1.03	0.063
Unknown	0.21	0.11-0.424	<0.001

*Stage IV and unknown were excluded from this analysis (n=464).
